# A MATE Transporter is Involved in Pathogenicity and IAA Homeostasis in the Hyperplastic Plant Pathogen *Pseudomonas savastanoi* pv. *nerii*

**DOI:** 10.3390/microorganisms8020156

**Published:** 2020-01-22

**Authors:** Stefania Tegli, Lorenzo Bini, Silvia Calamai, Matteo Cerboneschi, Carola Biancalani

**Affiliations:** 1 Dipartimento di Scienze e Tecnologie Agrarie, Alimentari Ambientali e Forestali, Laboratorio di Patologia Vegetale Molecolare, Università degli Studi di Firenze, Via della Lastruccia 10, 50019 Sesto Fiorentino (Firenze), Italy; lorenzo.bini5@stud.unifi.it (L.B.); silvia.calamai@unifi.it (S.C.); carola.biancalani@unifi.it (C.B.); 2 Next Genomics srl, Via Madonna del Piano, 6, 50019 Sesto Fiorentino (Firenze), Italy; matteo@nextgenomics.it

**Keywords:** *Pseudomonas savastanoi*, indole-3-acetic acid (IAA), IAA–Lysine, multidrug and toxic compound extrusion transporter, MATE, Type Three Secretion System (TTSS)

## Abstract

During the last years, many evidences have been accumulating about the phytohormone indole-3-acetic acid (IAA) as a multifaceted compound in the microbial world, with IAA playing a role as a bacterial intra and intercellular signaling molecule or as an effector during pathogenic or beneficial plant–bacteria interactions. However, pretty much nothing is known on the mechanisms that bacteria use to modulate IAA homeostasis, in particular on IAA active transport systems. Here, by an approach combining in silico three-dimensional (3D) structural modeling and docking, mutagenesis, quantitative gene expression analysis, and HPLC FLD auxin quantitative detection, for the first time a bacterial multidrug and toxic compound extrusion (MATE) transporter was demonstrated to be involved in the efflux of IAA, as well as of its conjugate IAA–Lysine, in the plant pathogenic hyperplastic bacterium *Pseudomonas savastanoi* pv. *nerii* strain *Psn23*. Furthermore, according to the role proved to be played by *Psn23* MatE in the development of plant disease, and to the presence of *Psn23* MatE homologs in all the genomospecies of the *P. syringae* complex, this membrane transporter could likely represent a promising target for the design of novel and selective anti-infective molecules for plant disease control.

## 1. Introduction

Auxin are plant hormones whose correct homeostasis is pivotal for proper plant growth and development, as well as for plant defense [1]. Indole-3-acetic acid (IAA) is the main and most abundant naturally occurring auxin in plants, as well as the best studied, whose *de novo* biosynthesis is mainly through four interlinked pathways having L-tryptophan (Trp) as a precursor. Generally, the Trp-dependent pathways are two-step reactions, named accordingly to their specific key intermediate molecule, specifically, indole-3-pyruvic acid (IPyA), indole-3-acetamide (IAM), tryptamine (TAM), or indole-3-acetaldoxime (IAOX). Less information is definitely available for Trp-independent IAA biosynthesis, where indole-3-glycerol phosphate or indole are considered the main precursors. The IPyA and IAM pathways are considered the most conserved and used routes for IAA biosynthesis in plants. However, many other important aspects still remain to be fully elucidated, such as which pathways are used in the different plant species and if they are likely to play alternative roles [2,3].

Firstly discovered in human urine and structurally similar to melatonin in animals [4], in addition to plants IAA is also produced by microalgae, archaea, bacteria, fungi, and yeasts [5]. Although the ability to synthesize IAA in bacteria and fungi is not restricted to those associated to plants, the role of microbial IAA in the interactions between plants and phytopathogenic or beneficial bacteria and fungi is the most studied [6].

Microbial IAA biosynthesis is strictly Trp-dependent, according to at least five different routes, including the IPyA and TAM pathways, as well as the tryptophan side-chain oxidase (TSO) pathway [7,8]. In gall- and tumor-forming bacteria and fungi, IAA has been shown pivotal for the development of hyperplastic symptoms, and its biosynthesis is generally through the IAM pathway. Conversely, the IPyA pathway is mainly represented in beneficial bacteria and fungi. Interestingly, the hyperplastic plant pathogenic bacterium *Pantoea agglomerans* has both the IPyA and IAM pathways, that are preferentially expressed during epiphytic colonization and the pathogenetic process, respectively [9,10].

Phylogenetic analysis carried out on key genes for IAA biosynthesis in organisms and microorganisms indicates that an independent but convergent evolution was occurred [5]. This finding strongly suggests a universal role of IAA as a signal molecule, both for the producers and during their biotic interactions at different taxonomic levels (e.g., intra and interspecies and even interkingdom) [11]. Plant pathogens have been demonstrated to produce IAA to hijack plant immunity, by subverting plant auxin signaling to increase host susceptibility to infection [6,12,13,14]. In addition, microbial IAA is also essential as signal molecules within the producer populations, and in plant pathogenic bacteria, IAA was demonstrated to affect the expression of genes of their virulence network [7,15,16,17].

However, the multiple effects triggered or dynamically modulated by IAA do not exclusively depend on its *de novo* biosynthesis. In plants, significant and coordinated changes occur over time for local IAA concentrations, as well as for its bioactive forms, also as a consequence of the IAA active polar transport throughout the whole plant and of other processes, such as its catabolism, conjugation, oxidation, storage, and even its signal transduction [18]. A similar fine and dynamic control of IAA homeostasis seems to occur also in bacteria, such as clearly demonstrated for the hyperplastic plant pathogen *Pseudomonas savastanoi* pv. *nerii*. Its ability to cause “knots” on its hosts relies on a functional Type Three Secretion System (TTSS), as well as on the bacterial IAA biosynthesis by the IAM pathway [19,20,21]. In addition, during the pathogenetic process, *P. savastanoi* pv. *nerii* regulates free IAA levels in the infected tissues by its conversion to the conjugate IAA–Lysine (hereafter indicated as IAA-Lys), supposed to be less biologically active than the IAA free form. This reaction is mediated by the enzyme IAA-Lys synthase, encoded by the *iaaL* gene [17]. Interestingly, most of the *P. syringae* pathovars and strains possess the *iaaL* gene in their genomes, even if they do not cause hyperplastic symptoms, and this gene appears to be very well conserved and present independently from the genes for IAA biosynthesis [22,23]. It is worth pointing out that the conversion of IAA to IAA-Lys is an exclusive trait of bacteria belonging to the *P. syringae* complex, and plants neither produce IAA-Lys nor are able to degrade it. Overall, these findings suggest for the bacterial conversion of IAA to IAA-Lys a widely conserved role in the dynamic regulation of the IAA content at and near the infection site. In this frame, it is thus not surprising that in *P. savastanoi* pv. *nerii* both the expression of the operon for IAA biosynthesis and that of *iaaL* gene are also under the control of TTSS, in addition to being auxin-regulated, to further stress the involvement of bacterial IAA and IAA-Lys in the plant–pathogen dialogue since the very first steps of their interaction [17].

Obviously, if a similar dialogue has to take place, it is reasonable to assume that bacterial IAA, and perhaps also its IAA-Lys conjugate, needs to be transported some way out of the bacterial cell into the apoplast. In the *P. savastanoi* pv. *nerii* strain *Psn23* genome, a gene coding a putative multidrug and toxic compound extrusion (MATE) efflux transporter (hereafter named *matE*) was found upstream to the *iaaL* gene, and whose expression was demonstrated to be TTSS-regulated [17]. MATE transporters are widely distributed in Gram-positive and Gram-negative bacteria, where they are usually associated with the efflux of organic cations for multidrug resistance. Conversely, MATE pumps found so far in plants have been demonstrated to be involved in the transport of a broader range of substrates than in bacteria, and having many other roles beyond detoxification, including the efflux of plant hormones and the regulation of plant disease resistance to pathogens, respectively [24,25].

The aim of this study was to analyze the structure of the putative MATE transporter identified in *Psn23* (hereafter named *Psn23* MatE) through the application of bioinformatics tools and to evaluate the role played by *Psn23* MatE in the development of plant disease and its relationship with the IAA efflux and homeostasis.

## 2. Materials and Methods

### 2.1. Bacterial Strains and Growth Conditions

The bacterial strains used in this study are listed in Table S1. *Pseudomonas savastanoi* pv. *nerii* strain *Psn23* and its mutants were routinely grown at 26 °C on King’s B (KB) [26] or *hrp*-inducing minimal medium (MM) [27], while *Escherichia coli* strains TOP10 and ER2925 were grown on Luria-Bertani (LB) [28], as liquid or agarized cultures. Bacterial growth in liquid media was monitored by measuring optical density (OD) at 600 nm (OD_600_) with a spectrophotometer (Infinite^®^ M200 PRO Multimode Reader, Tecan Group Ltd., Männedorf, Switzerland), while the concentration of viable bacteria was evaluated by plate counts and expressed as colony forming units per milliliter (CFU/mL). For long-term storage, bacteria were maintained at −80 °C on 40% (v/v) glycerol, and *P. savastanoi* cultures were periodically monitored by using specific PCR-based assays to exclude any bacterial contamination [29,30]. Antibiotics were added to growth medium if needed, and used at the following final concentrations: 20 µg/mL streptomycin, 50 µg/mL nitrofurantoin, 10 µg/mL gentamicin, and 50 µg/mL kanamycin.

### 2.2. Molecular Techniques

Unless otherwise stated, routine DNA manipulations and PCR were carried out using standard procedures [31] or according to manufacturers’ instructions. The plasmids used in this study are reported in Table S1. Genomic DNA from *P. savastanoi* strains was extracted from single bacterial colonies using thermal lysis [29], or from bacterial cultures (OD_600_ = 0.8), using Puregene^®^ Genomic DNA Purification Kit (QIAGEN, Hilden, Germany). DNA concentration was evaluated both spectrophotometrically with NanoDrop™ ND-1000 (NanoDrop Technologies Inc., DE, USA) and visually by standard agarose gel electrophoresis on 1% agarose (w/v) in TBE 1× [31]. For plasmid DNA extraction, NucleoSpin^®^ Plasmid (Macherey-Nagel GmbH & Co. KG, Düren, Germany) was used according to the manufacturer’s protocol. Amplicons were purified from agarose gel using NucleoSpin^®^ Gel and PCR clean-up (Macherey-Nagel GmbH & Co. KG) and then double-strand sequenced at Eurofins Genomics (Ebersberg, Germany). Primers were designed using Beacon Designer 7.7 software (Premier Biosoft International, Palo Alto, CA, USA), and their sequences and features are reported in Table S2.

### 2.3. Construction of *Psn23* Mutants for matE Gene

Five mutants were here produced from the wild-type strain *Psn23* [30] for the *matE* gene. The primers were designed according to the *matE* nucleotidic sequence of *Psn23* strain (GenBank Accession Number KU351686), and here used to generate and analyze the mutants listed in Table S2. The suicide vector for *P. syringae sensu lato* pK18-Δ*hrpA* [17,32] was used to clone the mutated *matE* constructs into *E. coli* cells, and then for their transfer into electrocompetent *Psn23* cells by using Gene Pulser XCell™ (Bio-Rad Laboratories Inc., Hercules, CA, USA) to replace the native *matE* gene by marker exchange [17]. A preliminary PCR screening of the putative *matE* mutants was carried out on transformed *Psn23* Suc^R^/Kan^S^ colonies, and then the marked mutations were confirmed by DNA sequencing. A stable knockout Δ*matE* mutant was constructed by an in-frame deletion of *matE* gene from the *Psn23* genome. Three alanine-substituted mutants for the putative *Psn23* MatE were also generated. The *matE* gene from *Psn23* was cloned into the *PstI* and *EcoRI* sites of pK18-Δ*hrpA* to produce the pK18*-matE* recombinant vector (Table S1). On this plasmid, the alanine substitutions D182A, Y200A, and T17035A were introduced into *matE* by using mutagenic PCR primers and QuickChange II Site-Directed Mutagenesis Kit (Agilent Technologies, La Jolla, CA, USA). The overexpressing mutant *Psn23*_pT3-*matE* was also constructed. The recombinant plasmid pT3*-matE* (Table S1) was produced starting from the vector pLPVM-T3A, which contains the native promoter of *hrpA* gene for *Psn23* [33]. The *matE* gene, amplified from pK18*-matE* using the primers pT3_matE_BamHI_For/pT3_matE_KpnI_Rev, was cloned into the *KpnI* and *BamHI* restriction sites of pLPVM-T3A and then electroporated into *Psn23* cells. 

### 2.4. *In Planta* Phenotypic Characterization of *Psn23* matE Mutants

Hypersensitive Response (HR) assay was carried out on 2 months old *Nicotiana tabacum* plants (var. Burley White), grown at 24 °C and at 75% relative humidity with a photoperiod of 16/8 h of light/dark. Bacterial cultures, grown overnight in KB medium at 26 °C, were washed twice in sterile physiological solution (SPS, 0.85% NaCl in distilled water) and then resuspended to reach an OD_600_ = 0.5. Bacteria were then injected into the mesophyll of fully expanded leaves of Tobacco plants, using a 2 mL blunt-end syringe pressed against the abaxial surface (approximately 100 μL/spot) [34]. The appearance of macroscopic symptoms associated to the development of HR was monitored in the next 48 h post-infiltration, taking photographic records of the results obtained. Pathogenicity trials with *Psn23* and its mutants were carried out on in vitro micropropagated oleander plants (var. Hardy Red), grown on Murashige–Skoog medium (MS) [35] without addition of any phytohormone for 3 weeks, at 26 °C and with a photoperiod of 16 h/light and 8 h/dark, as described previously [30,36]. Plants were periodically monitored for symptoms appearance, and the bacterial growth was estimated at 7, 14, and 21 days post inoculation (dpi). Three independent experiments were performed, and three plants for each *P. savastanoi* strain were used in each run.

### 2.5. Reverse Transcription-Quantitative PCR (qRT-PCR) and Gene Expression Analysis 

Liquid cultures of the wild-type strain *Psn23* and its mutants were grown overnight at 26 °C in KB, on an orbital shaker (100 rpm), then washed twice with SPS and transferred into MM medium supplemented with L-Trp (0.25mM) to reach an OD_600_ = 0.5. After 24 h at 26 °C and under shaking condition, bacterial cultures were collected and used for RNA extraction, performed with NucleoSpin^®^ RNA Plus (Macherey-Nagel GmbH & Co. KG), after a treatment with NucleoSpin^®^ gDNA Removal Column (Macherey-Nagel GmbH & Co. KG) to eliminate any genomic DNA. Then, RNA Reverse transcription was carried out on about 5 µg of total RNA per sample, by using iScript™ Advanced cDNA Synthesis kit (Bio-Rad Laboratories Inc.). Diluted cDNA was analyzed with SsoFast™ EvaGreen^®^ Supermix (Bio-Rad Laboratories Inc.), according to manufacturer’s protocols, using the CFX96 Real-Time PCR Detection System and CFX Manager software v1.6 (Bio-Rad Laboratories Inc.). The specific primers pairs here designed and used are listed in Table S2. The expression of each monitored gene was quantified using the 2^−ΔΔCt^ method, and the 16S rRNA gene for normalization. For each sample, three biological replicates were processed in each of the three independent qRT-PCR experiments here carried out. 

### 2.6. Quantification of Bacterial IAA Synthesis

The amount of IAA produced in vitro by the wild-type strain *Psn23* and its mutants was assessed both by the colorimetric Salkowski’s method [37] and by high-performance liquid chromatography coupled with fluorescence detection (HPLC FD). Bacterial cultures were grown overnight at 26 °C in KB on an orbital shaker (100 rpm), then washed twice with SPS and transferred into MM supplemented with L-Trp (0.25 mM). At 24 and 48 dpi, bacterial growth was recorded as OD_600_. The bacterial cultures were centrifuged in a microcentrifuge at 5,000 rpm for 10 min. The supernatants were collected, filter-sterilized on 0.2 μm pore size membranes (Sarstedt, Nümbrecht, German), and then used as such for IAA determination both by Salkowski’s assay and HPLC FD. Standards for IAA, L-Trp, and IAM at high purity grade (98%) for analytical applications were purchased from Sigma-Aldrich Co. (St. Louis, MO, USA), while IAA–Lysine was kindly synthesized by Department of Chemistry of the University of Padova, Italy. Standard curves were prepared by five ten-fold dilutions of each molecule, starting from 100 ppm in 35% MeOH. The HPLC analyses were performed on a HP 1100 Series chromatograph (Agilent Technologies, Waldbronn, Germany), equipped with diode array (DAD) and fluorescence (FLD) detectors. Chromatographic separations were carried out using a reverse-phase HPLC column ZORBAX ODS (4.6 mm x 250 mm; 5µm) (Agilent Technologies), whose temperature was set up at 40 °C. A 50 µl injection volume and flow rate at 0.9 mL/min were selected. Analytes were separated with an isocratic elution in 35% MeOH. The detection was performed in absorbance at 273 nm and in fluorescence using λ_ex_ 280 nm and λ_em_ 340 nm. The HPLC–DAD/FLD system control, as well as data acquisition and analysis were performed using the ChemStation A.10.01 software (Agilent Technologies).

### 2.7. Bioinformatic Analysis

Multiple sequence alignments and comparisons were performed by using the computer package Clustal Omega (https://www.ebi.ac.uk/Tools/msa/clustalo/) [38] and basic local alignment search tool (BLAST) (http://www.ncbi.nlm.nih.gov/blast) [39]. Phylogenetic analyses were carried out using neighbor-joining statistical method [40], and phylogenetic trees were generated in MEGA, version 7.0.18 [41]. Bootstrap analysis used 500 replications, Poisson model for substitutions and pairwise deletion method for data treatments (gaps). The cut-off value for condensed tree was 90%. According to the putative MATE protein from *Psn23*, three-dimensional (3D) structural models were produced by Phyre2 (http://www.sbg.bio.ic.ac.uk/phyre2/html/page.cgi?id=index) [42] and RaptorX (http://raptorx.uchicago.edu/) under default settings [43]. Molecular models visualization was performed using the software USCF Chimera (https://www.cgl.ucsf.edu/chimera/) [44]. The in silico prediction of ligand-binding sites for the interaction with target molecules was made by molecular docking, using the GEMDOCK software (BioXGEM) [45] and AutoDock Vina [46], and the molecular structure of L-Trp, IAM, IAA, and IAA–lysine were assembled in digital format using MarvinSketch 17.6 software (ChemAxon, Budapest, Hungary) (http://www.chemaxon.com).

### 2.8. Data Collection and Statistical Analysis

The experiments reported in this study were always carried out in triplicate, and at least three independent experiments were performed. The results are reported as means values ± standard deviation (SD). PAST software version 3.11 was used [47] to perform one-way ANOVA followed by Tukey–Kramer’s post-test analysis, and *p* values ≤ 0.05 were considered to be statistically significant. 

## 3. Results

### 3.1. *In Vitro* IAA Production by *Psn23* Depends from a Functional matE Gene

**Figure 1:**
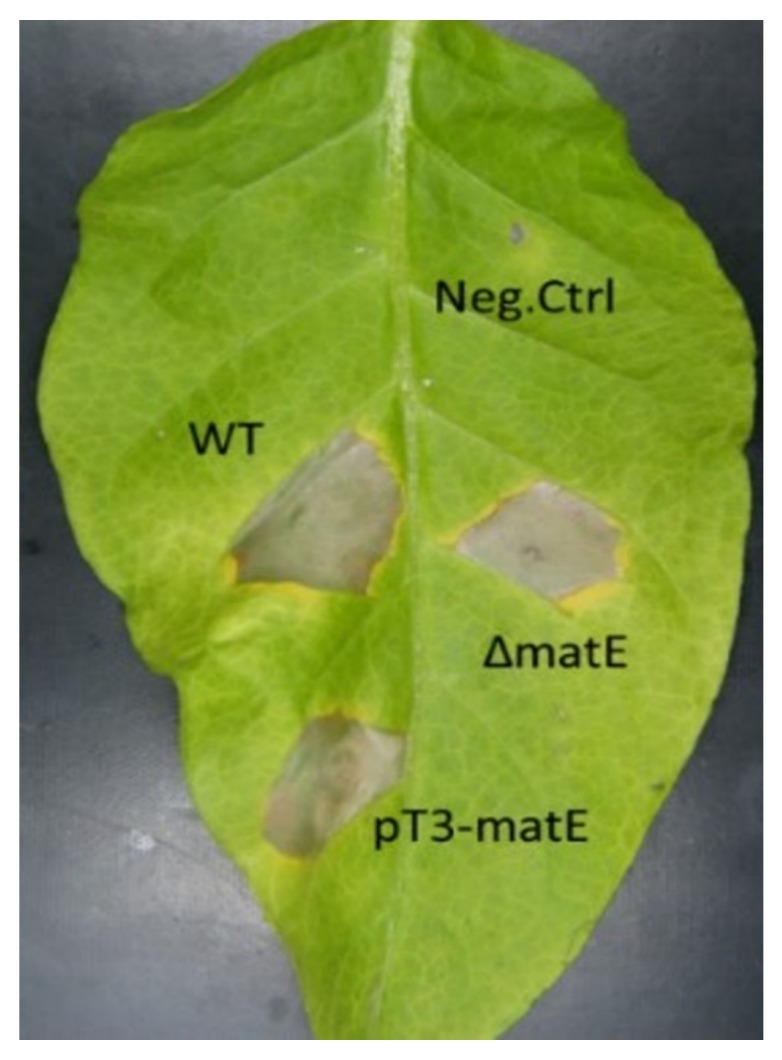
Hypersensitive Response assay on *N. tabacum*. Tobacco leaves were infiltrated with bacterial suspensions of Δ*matE* and pT3-*matE* mutants. For comparison, the wild-type *Psn23* (WT) was also tested. As negative control, sterile physiological solution was used (Neg.Ctrl). Picture was taken 48 h post-infiltration.

Upstream to the gene *iaaL*, an ORF encoding a putative MATE efflux transporter (GenBank AOR51355) was found near the *iaaM*/*iaaH* operon in *P. savastanoi* pv. *nerii Psn23*, having a TTSS-dependent expression and hypothesized to mediated IAA efflux, in addition to confer resistance to drugs such as 8 hydroxyquinoline [17]. To test this hypothesis, the in-frame deleted mutant Δ*matE* was produced. As a control, the overexpressing mutant pT3*-matE* also was generated. Here, the *matE* gene was under the control of an inducible promoter, that is, the promoter driving the expression of *hrpA* in the TTSS of *Psn23*, hereafter named pT3. This promoter can be switched on in vitro when *Psn23* is grown on minimal medium (MM), mimicking the apoplast conditions [27]. No significant differences were observed between the in vitro growth of *Psn23* and its mutants Δ*matE* and pT3*-matE*, when incubated at 26 °C on MM or on KB, during the first 72 h in shaking condition (100 rpm) (data not shown). Similarly, no differences were found in the ability of the wild-type strain *Psn23* and of its mutants Δ*matE* and pT3*-matE* to cause HR after their infiltration into the mesophyll of Tobacco leaves (Figure 1).

**Figure 2:**
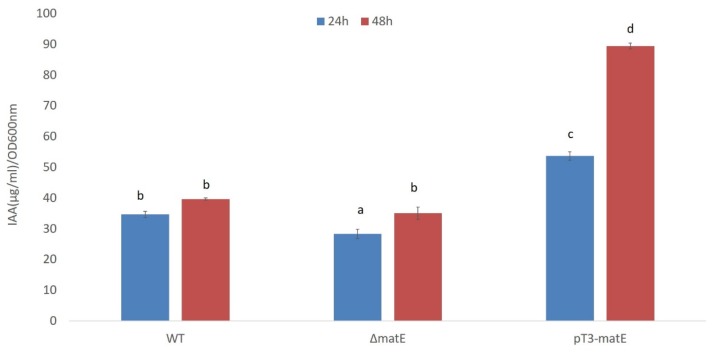
IAA in vitro production by *Psn23* and its Δ*matE* and pT3-*matE* mutants. Bacteria were grown on minimal medium (MM) supplemented with L-tryptophan (Trp, 0.25mM), at 26 °C in shaking condition. Salkowski’s assay was carried out on bacterial supernatants collected after 24 h (blue) and 48 h (red) of growth. The data represent the average of three independent experiments, each with replicates ± standard deviation (SD). Different letters indicate significant differences among means of mutants at *p* < 0.05, according to Tukey’s test.

Conversely, strongly statistically significant differences were found between *Psn23* and its mutants as far as the in vitro production of IAA is concerned. After 24 and 48 h of incubation at 26 °C on MM supplemented with Trp, the concentration of IAA in the bacterial free culture supernatants was evaluated by the colorimetric assay based on Salkowski’s reagent [17,48]. As shown in Figure 2, after 24 h the amounts of IAA released into the culture medium by the mutants Δ*matE* and pT3-*matE* were significantly lower and higher, respectively, than that of the wild-type *Psn23*. The exceptionally increased value in IAA production obtained for the mutant pT3*-matE* was then confirmed as statistically significant also after 48 h. 

### 3.2. Expression of *matE* Gene Influences Expression of Genes for IAA Production and Pathogenicity

**Figure 3:**
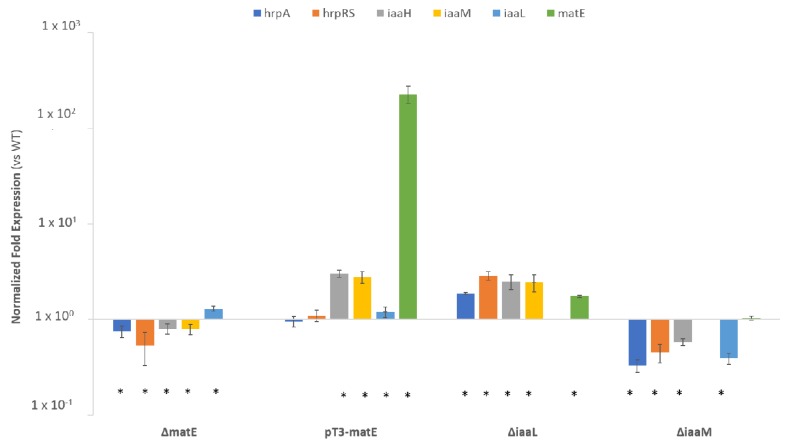
In vitro gene expression analysis of the *Psn23* mutants Δ*matE*, pT3-*matE*, Δ*iaaL,* and Δ*iaaM.* Bacteria were grown in vitro on MM for 24 h, and their gene expression was compared with that of the wild-type *Psn23*, for the genes *hrpA* (light blue), *hrpRS* (orange), *iaaH* (grey), *iaaM* (yellow), *iaaL* (blue), and *matE* (green). Data are averages of triplicates ± standard deviation (SD). Asterisks indicate significant differences compared with the untreated sample at *p* < 0.05.

The expression of genes related to IAA biosynthesis (*iaaM* and *iaaH*) and metabolism (*iaaL*), to the activation of the master pathogenicity system TTSS (*hrpRS* and *hrpA*) as well as to the putative *Psn23* MatE transporter here studied (*matE*), was then evaluated by real-time PCR on *Psn23* and its *matE* mutants, grown on MM supplemented with Trp. The mutants Δ*iaaM* and Δ*iaaL* were also included for comparison. The expression of *iaaM* and *iaaH* was statistically significantly reduced in the mutant Δ*matE* in comparison with the wild-type *Psn23*, while it was upregulated in the overexpressing mutants pT3*-matE* (Figure 3). These results are in accordance with the data obtained on the in vitro IAA production for the same bacteria, and with the feedback inhibition of IAA on its own biosynthesis, as known for *Psn23* [17].

Interestingly, in the mutant Δ*matE,* also the genes related to the TTSS were downregulated, and just the gene *iaaL* appeared to be overexpressed in comparison with the wild-type *Psn23*. The gene expression profile of the Δ*matE* mutant resulted here quite close to that of Δ*iaaM* mutant, with the exception of the gene *iaaL.*

Similarly to the pT3*-matE* mutant, the genes for IAA biosynthesis and *matE* were statistically significantly overexpressed in the Δ*iaaL* mutant. Overall, these findings strongly suggest the involvement of the putative *Psn23* MatE membrane protein also in the IAA homeostasis of this bacterium, in particular to mediate IAA efflux.

### 3.3. Virtual 3D Modelling of *Psn23* MatE, and Prediction of IAA and IAA-Lys as Putative Substrates

**Figure 4:**
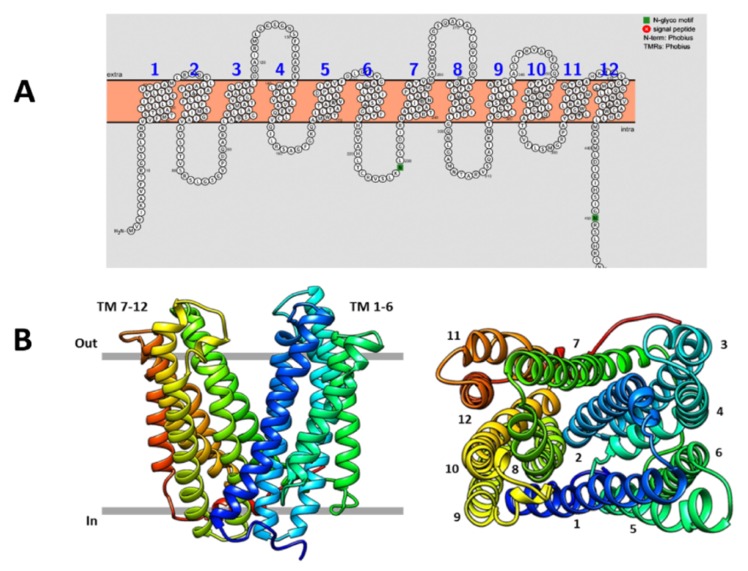
Predicted membrane topology and structure of the *Psn23* MatE transporter. (**A**) Twelve putative transmembrane (TM) domains were predicted for *Psn23* MatE, on the basis of amino acid hydrophobicity; (**B**) Ribbon three-dimensional (3D) model of *Psn23* MatE, viewed parallel to the membrane (left) and along the membrane normal from the periplasmic side (right), with the twelve TM helices numbered starting from the first N amino acid (methionine).

Concerning microbial IAA, up to now just the fungal MATE transporter Mte1 of *Tricholoma vaccinum* has been demonstrated to have a role in IAA efflux, by an indirect approach based on the use of the IAA transport inhibitor 2,3,5-triiodobenzoic acid [49]. Unfortunately, the crystal structure of Mte1 is not yet available to perform the most appropriate structure–activity studies to unequivocally demonstrate its involvement in IAA active transport. Conversely, the crystal structures of some bacterial MATE transporters are already available, and all of them are membrane proteins characterized by the presence of twelve transmembrane helices (TM 1–12), forming an internal cavity. In this pocket, several quite specific and conserved residues provide the binding sites for the substrates and for those ions (H^+^ or Na^+^) whose gradient across the membrane serves as energy source [50]. As shown in Figure 4, also the putative *Psn23* MatE transporter consists of twelve TMs arranged as two bundles (TM 1–6 and TM 7–12), one at the N- and the other at the C-terminal domain, forming the above-mentioned pocket with a typical V-shaped conformation. 

According to this 3D model, in the TM1 and TM5 of the N lobe of *Psn23* MatE two negative amino acids (E35 and D182) were found to be located, as occurring in the same TMs (D41 and D184) of the H^+^-driven MATE transporter from *Pyrococcus furiosus* (known as *Pf*MATE) to give the cation-binding site [51,52]. In contrast, no negative-charged amino acids were found in the TM7 and TM10 of *Psn23* MatE as well as of *Pf*MATE, as occurring in the NorM-type MATE transporters which usually use the Na^+^ motive force to drive the substrate transport across the membrane [52,53].

Overall, these findings support the hypothesis of *Psn23* MatE transporter belonging to the DinF subfamily of the prokaryotic MATE transporters. On these bases, a virtual structure-based ligand analysis was then performed to assess if IAA and its conjugate IAA-Lys could be substrates for *Psn23* MatE, by using the GEMDOCK software (BioXGEM) and *Pf*MATE as comparative protein structure model [45]. The binding free energy calculations showed that free IAA and IAA-Lys could be realistically considered among the potential substrates transported by *Psn23* MatE across the bacterial membrane, and that the Y200, T170, T173, and T175 residues are important in substrate binding (Table 1). 

While Y200 has to be considered potentially involved in the binding of both free IAA and of its conjugate with Lysine, the residues T170, T173, and T175 showed to have a stronger interaction with IAA-Lys than with IAA, probably because of a specific affinity of polar uncharged amino acids for lysine, such as occurring for threonine.

### 3.4. Site-Directed Mutagenesis of *Psn23* MatE to Confirm Its Involvement in Pathogenicity and IAA Secretion

According to the data from virtual 3D modelling and docking analysis performed on *Psn23* MatE, site-directed mutagenesis was then carried out on those residues of its N-lobe supposed to be involved in cation-binding (*i.e*., D182) or in substrate recognition and interaction (*i.e*., T170, T173, T175, and Y200). Therefore, the alanine-substituted mutants D182A and Y200A were obtained by single substitution events, while by a triple substitution the mutant T17035A was generated. As previously reported for the *Psn23* mutants Δ*matE* and pT3*-matE*, the in vitro growth of the alanine-substituted mutants D182A, Y200A, and T17035A was not impaired in comparison with the wild-type *Psn23*, when incubated at 26 °C on MM or on KB in shaking condition (data not shown).

Pathogenicity tests were then carried out on these mutants, as well as on the Δ*matE* and pT3*-matE* mutants, by using in vitro micropropagated oleander plants (var. Hardy Red) [17]. For comparison, the wild-type *Psn23* was also used. Plants were periodically monitored for symptoms appearance, and the development of the typical hyperplastic knot was firstly visible at naked eyes at about 7 dpi. The *in planta* bacterial growth was also periodically evaluated at 7, 14, and 21 dpi.

**Figure 5:**
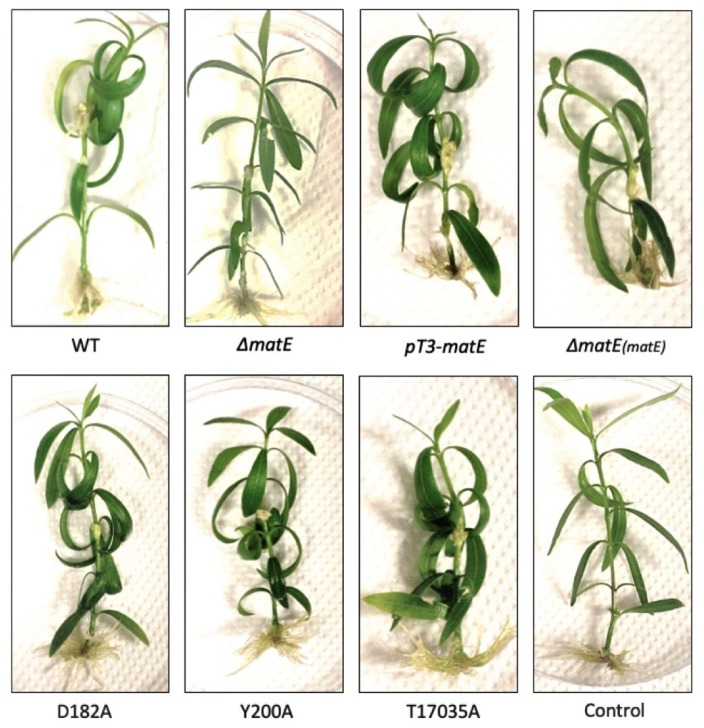
Hyperplastic symptoms obtained at 21 dpi in pathogenicity tests carried out on micropropagated oleander plants with *Psn23* wild type and its mutants Δ*matE*, pT3*-matE*, D182A, Y200A, and T17035A. Control: sterile physiological solution (SPS, NaCl 0.85%) inoculated plants. Complemented mutant for Δ*matE*: Δ*matE*(*matE*).

The results obtained at 21 dpi are shown in Figure 5, where it is obvious the significant reduction in the size of the knots occurring in the Δ*matE-*inoculated plants, as well as on those infected by the alanine-substituted mutants D182A and Y200A.

**Figure 6:**
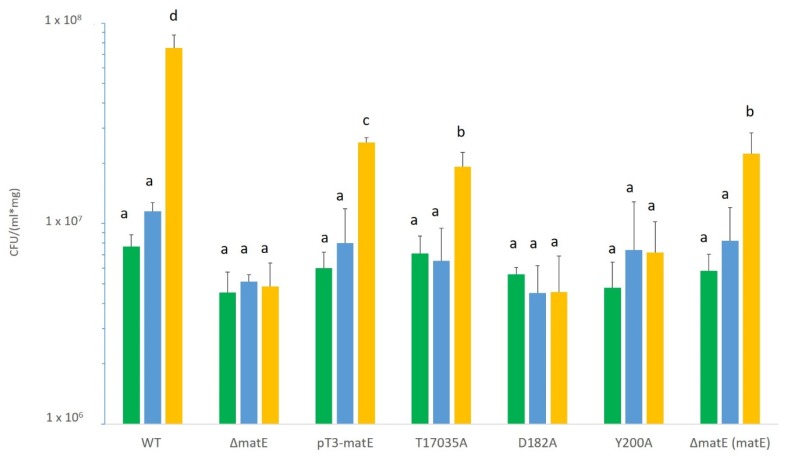
*In planta* bacterial growth of *Psn23* wild type and its mutants Δ*matE*, pT3*-matE*, D182A, Y200A, and T17035A, at 7 (green), 14 (blue), and 21 (yellow) dpi. Values are the mean of three independent experiments, with nine replicates for run and for each strain ± standard deviation (SD). Different letters indicate statistically significant differences among means at *p* < 0.05, according to Tukey’s test. Complemented mutant for Δ*matE*: Δ*matE*(*matE*).

Their hypovirulent phenotype was further confirmed by the data on their growth *in planta*, which was statistically significantly reduced in comparison with the wild-type *Psn23* (Figure 6). A reduction in their *in planta* growth ability was also observed at 21 dpi for the mutants T17035A and pT3*-matE*, although the hyperplastic galls they generated on infected plants were slightly bigger or comparable in size to those for the wild-type *Psn23*, for pT3*-matE* and T17035A, respectively (Figure 6).

**Figure 7:**
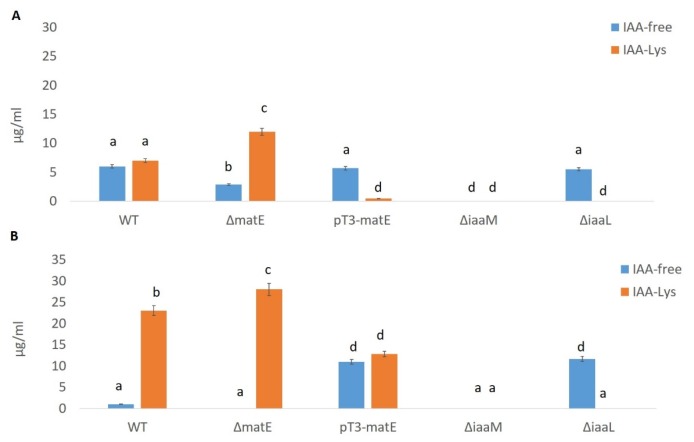
IAA in vitro production by the wild-type *Psn23* (WT) and its mutants Δ*matE*, pT3*-matE*, Δ*iaaM*, and Δ*iaaL.* After 24 h (**A**) and 48 h (**B**) of in vitro growth on MM supplemented with Trp, free IAA and IAA-Lys were quantified in the bacterial supernatants by HPLC–FLD. Data are averages of triplicates ± standard deviation (SD). Different letters indicate significant differences among means of mutants at *p* < 0.05, according to Tukey’s test.

These findings undoubtedly demonstrated that the putative MATE transporter coded in *Psn23* by the *matE* gene is definitely involved in the virulence of this plant pathogen, expressed as ability to cause symptoms and to grow inside the infected plant host tissues. Most importantly, this role appears to depend on *Psn23* MatE ability to transport bacterial biosynthesized IAA using the H^+^ motive force, similarly to *Pf*MATE [52]. It was therefore essential to increase the resolution of the analysis for the quantification of IAA and its conjugate IAA-Lys, as well as its intermediate IAM, synthesized in vitro by *Psn23* and its MatE mutants by using high-performance liquid chromatography coupled with a fluorescent detector (HPLC–FLD). In Figure 7, the data related to the in vitro production of IAA and IAA-Lys are evaluated by HPLC–FLD on the cell-free filtrates of *Psn23* and the mutants Δ*matE* and pT3*-matE*, after 24 and 48 h of growth on MM supplemented with Trp. For comparison, the hypovirulent Δ*iaaM* and the hypervirulent Δ*iaaL* mutants were also tested, thus to confirm what was already known and expected, that is the Δ*iaaM* inability to synthesize IAA as well as the hyperproduction of free IAA obtained by Δ*iaaL.* The mutants Δ*matE* and pT3*-matE* have been here shown to have a behavior similar to that of Δ*iaaM* and Δ*iaaL*, respectively, for both IAA production and virulence.

**Figure 8:**
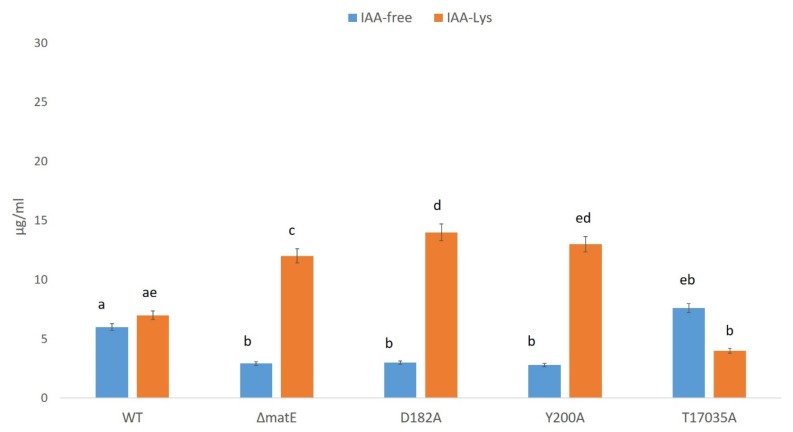
IAA in vitro production by the wild-type *Psn23* (WT) and its MatE alanine-substituted mutants D182A, Y200A, and T17035A. For comparison, the mutant Δ*matE* was also used. After 24 h of in vitro growth on Minimal Medium MM supplemented with Trp, free IAA and IAA-Lys were quantified in the bacterial supernatants by HPLC–FLD. Data are averages of triplicates ± standard deviation (SD). Different letters indicate significant differences among means of mutants at *p* < 0.05, according to Tukey’s test.

As far as the *Psn23* MatE alanine-substituted mutants are concerned, in Figure 8 the results obtained are reported. As expected, the hypovirulent mutants D182A and Y200A showed a reduced production of IAA and increased levels of IAA-Lys, with a behavior coherent to that of Δ*matE*, that is the other hypovirulent mutant. Accordingly, no particular differences were found in the biosynthesis of IAA and its metabolite for the T17035 mutant in comparison with the *Psn23* wild type, as occurring also for its ability to cause symptoms on the host plant.

## 4. Discussion

In plants, phytohormones are known to finely regulate plant morphogenesis and development, and their involvement in plant–microbe interactions has been demonstrated as well [1]. In this frame, a pivotal role is played by the auxin IAA, whose levels *in planta* can be modulated by phytopathogens to promote susceptibility in their potential hosts. Gram-negative phytopathogenic bacteria belonging to the so called *P. syringae* complex have been shown to hijack IAA accumulation or auxin signaling by specific virulence factors, such as several TTSS effectors. In addition, most *P. syringae* bacteria produce IAA, even if they do not cause any hyperplastic symptoms, and its role as a signaling molecule able to regulate bacterial gene expression as well as virulence has been ascertained [9,10]. The hyperplastic activity of *P. savastanoi* pv. *nerii* strain *Psn23* on oleander was shown to depend on the balance between free IAA and its conjugate IAA-Lys in the infected tissues, and this process was demonstrated to be under the control of TTSS [17]. In this auxin-based dialogue with plants, up to now the aspect definitely less investigated has been how IAA-producing phytopathogenic bacteria secrete this phytohormone into the apoplast.

Here, for the first time, a bacterial MATE transporter was demonstrated to mediate IAA efflux in the plant pathogen *P. savastanoi* pv. *nerii* strain *Psn23*, as already known for some plant MATE membrane proteins which mediate transport of several phytohormones, including auxins [24]. The same role has been demonstrated just for a microbial MATE, that is Mte1 from *T. vaccinum* [49]. By targeted mutagenesis, several amino acid residues involved in *Psn23* MatE functionality have been identified. According to these data and to its putative structure, obtained by in silico 3D modeling, *Psn23* MatE appears to belong to the DinF subfamily of the prokaryotic MATE transporters, such as the H^+^-driven MATE transporter from *P. furiosus* [51].

Just one other bacterial MATE transporter has been identified so far in plant pathogenic bacteria, particularly in *Erwinia amylovora*. The *norM* gene from *E. amylovora* codes for a protein highly homologous to the NorM MATE transporter of *E. coli* and *Vibrio parahaemolyticus*, and it was demonstrated to confer resistance to toxins produced by several epiphytic bacteria colonizing the same habitat in addition to the canonical resistance to some hydrophobic cationic antibiotics [54].

Here, for the first time, a bacterial MATE transporter was demonstrated to be involved in the molecular dialogue between a phytopathogenic bacterium and its potential host plant, by modulating IAA homeostasis in *Psn23* through the MATE-mediated auxin transport. It is well established that in *P. savastanoi* the expression of several TTSS genes is downregulated by IAA. Therefore, in the first step of the infection process induced by *P. savastanoi* it is reasonable to hypothesize that the intracellular IAA levels have to be carefully modulated by some homeostatic mechanisms, for the most part unknown but which certainly include IAA conjugation, to give auxin metabolites having a lower biological activity than IAA, such as IAA-Lys. In the Δ*iaaL* mutant, unable to conjugate IAA with Lysine, IAA secretion is strongly increased in association with a hypervirulent phenotype [17]. The same phenotype was here found for the overexpressing mutant pT3-*matE*. Conversely, a hypovirulent phenotype was scored for the *Psn23* mutants having their MATE transporter somehow impaired, as occurring for the deleted mutant Δ*matE* and for the alanine-substituted mutants D182A and Y200A. In addition, these mutants also showed a reduced in vitro IAA production in comparison with the wild-type *Psn23*. Overall, these data demonstrated that *Psn23* MatE mediates IAA efflux, thus to contribute to maintain the most appropriate IAA intracellular concentrations in each step of the whole infective process, as also suggested by its TTSS-dependent expression, in order to maximize the chances of success. This regulation of IAA homeostasis is played together with IAA-Lysine synthase. The *iaaL* expression is also TTSS-dependent, as well as coordinated with that of *matE* and IAA-inducible [17]. According to the experimental data obtained by HPLC–MS on the IAA in vitro production by *Psn23* and its MATE-mutants, *Psn23* MatE can be fairly hypothesized to transport IAA but not its conjugate IAA-Lys, for which additional secretion mechanisms have to be taken into consideration.

However, in addition to the virtual structure-based ligand analysis here carried out, the crystal structure determination and analysis of *Psn23* MatE is the next key step to definitely elucidate those substrates specifically recognized and transported by this membrane protein, as well as the actual antiporter cation (H^+^ or Na^+^). A deeper understanding of mechanisms underlying the physiology and the activity of these evolutionarily conserved transport proteins also in bacterial plant pathogens is advisable. For its role in the efflux of a virulence factor such as IAA and also in the resistance to several antimicrobials [17], *Psn23* MatE has indeed to be considered an important promising target for the development of innovative and ecofriendly strategies for the control of *Psn23* as well as of other pathogenic bacteria, possibly by the use of natural indole-mimicking competitors.

## Figures and Tables

**Table 1:** Energy values of interaction between selected *Psn23* MatE amino acids and several putative ligands. The value for each amino acid residue represents the energy of the single bond (expressed as kcal/mol). H = hydrogen bonding; V = van der Waals forces.

Ligand	Energy	H T175	H Y200	V T170	V T173	V T175	V Y200
**L-Trp**	−72.1	0	0	0	0	0	−11.1
**IAM**	−71.2	−2.9	−1.4	0	0	−0.9	−10.6
**IAA-free**	−102.5	0	0	0	0	−1.5	−28.5
**IAA-lysine**	−95.1	−5.9	0	−2.1	−3.5	−3.9	−16.8

## Supplementary Materials

The following are available online at https://www.mdpi.com/2076-2607/8/2/156/s1, Table S1: Bacterial strains, mutants, and plasmids used in this study; Table S2: Primers used in this study.
