# Supplementary material for: A MATE Transporter is Involved in Pathogenicity and IAA Homeostasis in the Hyperplastic Plant Pathogen Pseudomonas savastanoi pv. nerii
Source: Microorganisms. 2020 Jan 22;8(2):156. doi: 10.3390/microorganisms8020156 (PMC7074806; doi:10.3390/microorganisms8020156)
Supplement: Supplementary file 1 [file microorganisms-08-00156-s001.pdf]

Table S1. Bacterial strains, mutants and plasmids used in this study.

Strain/Plasmid	Relevant characteristics [a]	Reference/Source [b]
**Strain**		
E. coli TOP10	F-, mcrA, Δ(mrr-hsdRMS-crBC) Φ80 lacZΔM15 ΔlacX74 recA1 araD139 Δ(araleu)7697 galU galK rpsL (StrR) endA1 nupG	Invitrogen, Carlsbad, USA
E. coli ER2925	ara-14 leuB6 fhuA31 lacY1 tsx78 glnV44 galK2 galT22 mcrA dcm-6 hisG4 rfbD1 R(zgb210::Tn10)TetS endA1 rpsL136 dam13::Tn9 xylA-5 mtl-1 thi-1 mcrB1 hsdR2	NEB, Hertfordshire, UK
P. savastanoi pv. nerii (Psn23)	Wild type	LPVM collection [30]
Psn23_ΔiaaM	*iaaM* in-frame deletion mutant of *Psn23*	[17]
Psn23_ΔiaaL	*iaaL* in-frame deletion mutant of *Psn23*	[17]
Psn23_ΔmatE	*matE* in-frame deletion mutant of *Psn23*	This study
Psn23_pT3-matE	Gm^R, lacZ, mcs, *hrpA*_promoter+*matE*	This study
Psn23_D182A	*matE* replaced mutant (Asp182Ala) of *Psn23*	This study
Psn23_Y200A	*matE* replaced mutant (Tyr200Ala) of *Psn23*	This study
Psn23_T17035A	*matE* replaced mutant (Thr170Ala, Thr173Ala and Thr175Ala) of *Psn23*	This study
**Plasmid**		
pK18-ΔhrpA	pK18mobsacB derivative, in-frame deletion of the *hrpA* gene (273 bp), Km^R	[17]
pK18-*ΔmatE*	pK18mobsacB derivative, in-frame deletion of the *matE* gene (1113 bp), Km^R	This study
pK18-*matE* (D182A)	pK18mobsacB derivative, *matE* gene replaced in amino acid position 182 (Asp → Ala), Km^R	This study
pK18-*matE* (Y200A)	pK18mobsacB derivative, *matE* gene replaced in amino acid position 200 (Tyr → Ala), Km^R	This study
pK18-*matE* (T17035A)	pK18mobsacB derivative, *matE* gene replaced in amino acid positions 170, 173 and 175 (Thr → Ala), Km^R	This study
pLPVM-T3A	Gm^R, lacZ, mcs, *hrpA*_promoter+*gfp*	[33]
pLPVM-matE	Gm^R, lacZ, mcs, *hrpA*_promoter+*matE*	[33]

[a] Gm^R, gentamicin resistance; Km^R, kanamycin resistance. [b] LPVM Laboratorio di Patologia Vegetale Molecolare (University of Florence).

Table S2. Primers used in this study.

Primer name	Primer sequence (5′-3′) [a]
matE_PstI_For	TTTCTGCAGTCCAGAACACAGACATTT
matE_cross_Rev	CCGGATCCACTGAAACTTGCTATGCCAAGAATCATC
matE_cross_For	AAGTTTCAGTGGATCCGGCGAGATCCATTCAATAGG
matE_EcoRI_Rev	TTTGAATTCTAATCGTGTTGTTCAGAG
pT3_matE_BamHI_For	TTTGGATCCATGGTAGTTATCAA
pT3_matE_KpnI_Rev	AAAGGTACCTTATGAGTTACTCCTGT
pK18_matE_PstI_For	TTTCTGCAGATGGTAGTTATCAA
pK18_matE_EcoRI_Rev	TTTGAATTCTTATGAGTTACTCCTGT
matE_D182A_For	GTTGCCATCGCAGCCCCGCTGCTTATTG
matE_D182A_Rev	CAATAAGCAGCGGGGCTGCGATGGCAAC
matE_Y200A_For	CGGCATCGCCGCCCTGATATCGAG
matE_Y200A_Rev	CTCGATATCAGGGCGGCGATGCCG
matE_T17035A_For	GTGGGCCCTGCTGGCGGCGGCGGC
matE_T17035A_Rev	GCCGCCGCCGCCAGCAGGGCCCAC
hrpA_ RT_ For	GCAGGGTATCAACAGCGTCAAG
hrpA_ RT_ Rev	CCGTTCTCTTCGTTCGCAGTG
hrpRS_RT_For	ACCCGCAGAGTGAAGAAC
hrpRS_RT_Rev	CGCTTGAGTGACTGTTGAATC
iaaM_ RT_ For	TTCACTGCCTCACGGATAGCG
iaaM_ RT_ Rev	CGACTGGATGGTGGTGGGAAG
iaaL_ RT_For	ACCTCAGCAGCGGCGTAAAG
iaaL_ RT_ Rev	TCGTCGGTGTGTATGGCAGTTC
iaaH_RT_For	TGATGATGCCGATATTGTC
iaaH_RT_Rev	AAGGTGGTGATTGATGATG
matE_RT_For	CATCGCAGCCATTACG
matE_RT_Rev	AGCCTGAAGAACCTGTC

[a] The nucleotides underlined refer to digestion cutting sites for molecular cloning.
